# Outbreak of Listeriosis Likely Associated with Baker’s Yeast Products, Switzerland, 2022–2024

**DOI:** 10.3201/eid3011.240764

**Published:** 2024-11

**Authors:** Roger Stephan, Jule Anna Horlbog, Magdalena Nüesch-Inderbinen, Nicola Dhima

**Affiliations:** Institute for Food Safety and Hygiene, Zurich, Switzerland (R. Stephan, J.A. Horlbog, M. Nüesch-Inderbinen); National Centre for Enteropathogenic Bacteria and *Listeria*, Zurich (J.A. Horlbog, M. Nüesch-Inderbinen); Swiss Federal Office of Public Health, Bern, Switzerland (N. Dhima)

**Keywords:** listeriosis, bacteria, food safety, Listeria monocytogenes, ST3141, baker’s yeast, outbreak, foodborne disease, Saccharomyces cerevisiae, production site, contamination, Switzerland

## Abstract

We traced back a nationwide outbreak of human listeriosis in Switzerland to a persisting production line contamination of a factory producing baker’s yeast with *Listeria monocytogenes* serotype 1/2a sequence type 3141. We used whole-genome sequencing to match clinical isolates to isolates from product samples.

We report a prolonged and diffuse outbreak of listeriosis in Switzerland that involved 34 patients. The first case was reported in April 2022, and the outbreak peak occurred in 2023 (Figure 1). The last known case was in June 2024. A questionnaire-based outbreak investigation was initiated by the Swiss Federal Office of Public Health but did not identify a specific food exposure. The median age of the patients was 79 years (range 0–93 years); 18 (53%) were female and 16 (47%) male. The distribution of patients within Switzerland was wide (across different cantons) (Appendix Figure). 

**Figure 1:**
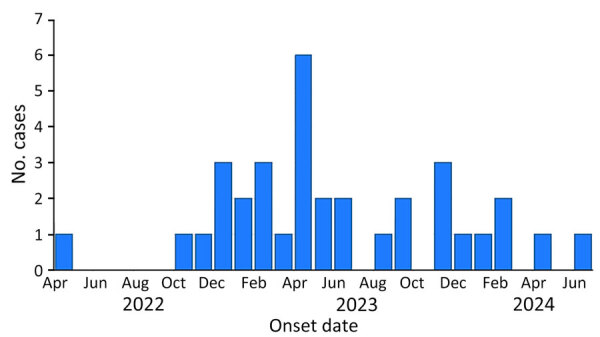
Listeriosis cases (N = 34), by illness onset date, in an outbreak likely linked to baker’s yeast products, Switzerland, April 2022–June 2024. The outbreak involved single distinct core-genome multilocus sequence typing cluster type 18049 of *Listeria monocytogenes* serotype 1/2a, sequence type 3141.

Of the 34 persons with documented cases, 7 died, and listeriosis was reported as the primary cause of death in all 7 cases according to the notification data. Of the 34 human *Listeria monocytogenes* isolates recovered, 28 were from blood, 2 from cerebrospinal fluid, and 1 from a swab specimen of a facial skin lesion; for 3 isolates, no information about the clinical sample was available. 

We performed whole-genome sequencing (WGS) by using Illumina MiSeq next-generation sequencing technology (Illumina, https://www.illumina.com). We mapped sequencing reads against a multilocus sequence typing scheme based on 7 housekeeping genes and a 1,701-locus core genome multilocus sequence typing (cgMLST) scheme by using Ridom SeqSphere+ software version 9.0.2 (https://www.ridom.de/seqsphere) (*1*). We determined sequence types and cluster types upon submission to the *L. monocytogenes* cgMLST Ridom SeqSphere+ server (*1*). 

We defined a cluster as a group of isolates with <10 different alleles between neighboring isolates (*1*). We assigned all 34 isolates to *L. monocytogenes* serotype 1/2a, sequence type 3141, cgMLST cluster type 18049; all isolates harbored *fosX* genes (coding for fosfomycin resistance) and *vga*(G) genes (coding for lincosamides and streptogramin A resistance). Furthermore, all isolates were genetically closely related (<2 allelic difference) to *L. monocytogenes* isolated from baker’s yeast (*Saccharomyces cerevisiae*) products from a commercial yeast factory and its production lines. 

The baker’s yeast products were sold in retail stores and supplied the food industry throughout Switzerland. During 2022, the baker’s yeast manufacturer had determined the occurrence of *L. monocytogenes* in yeast product samples. Analysis had been conducted as part of the manufacturer’s self-control practices; however, in the absence of legal requirements for non–ready-to-eat (non-RTE) products, the findings were not reported. In July 2023, official random and risk-based food checks performed by a cantonal laboratory in Switzerland revealed the presence of *L. monocytogenes* (isolate N23-1507) in a yeast product. WGS analysis confirmed the presence of the outbreak strain (Figure 2). The finding prompted extensive environmental sampling on the production site of the manufacturer and retrospective WGS analysis of all available isolates.

**Figure 2:**
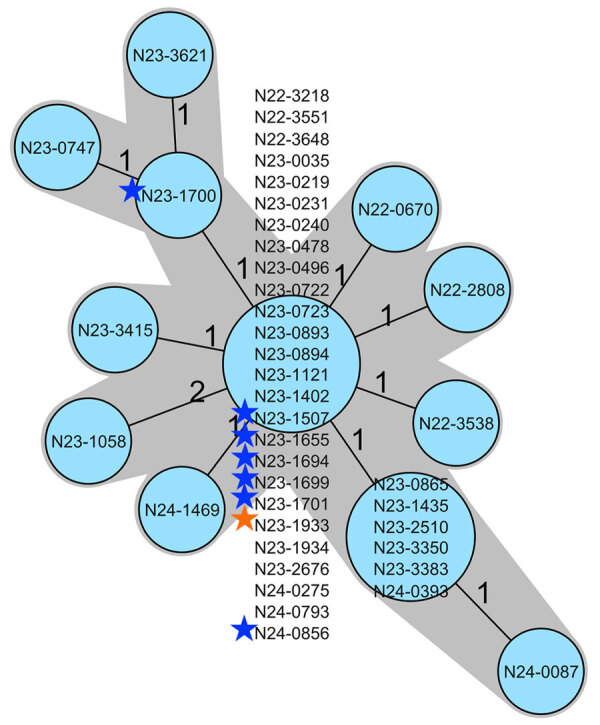
Minimum-spanning tree based on core-genome multilocus sequence typing allelic profiles of 34 human infections by *Listeria monocytogenes* sequence type 3141, a representative selection consisting of 7 baker’s yeast product isolates, and 1 isolate from a flow pipe in the baker’s yeast production line, Switzerland, 2022–2024. Each circle represents an allelic profile based on sequence analysis of 1,701 core-genome multilocus sequence typing target genes. Values on connecting lines indicate number of allelic differences between 2 strains. Each circle contains the identification of the strain or strains. Blue stars indicate the yeast product isolates; orange star indicates the environmental strain. Strain N23-1507 was isolated from baker’s yeast product during an official food check.

During July 2023–August 2024, the manufacturer commissioned microbiological testing on different production steps, and we obtained product samples or swabs from the yeast cream, vats, pipe systems, drying filters, extruders, conveyor belts, and cutting machines. All sequenced *L. monocytogenes* isolated from the production line and the strains obtained from yeast products matched the outbreak strain (Figure 2). Within the production line, the laboratory identified a flow pipe connecting a starch vat to the yeast drying bed as the first point of contamination. Subsequently, the manufacturer started deep cleaning of all processing equipment at the part of the production line where the positivity was detected. An official product recall was not warranted because baker’s yeast is not classified as RTE food. However, only batches of the product that had been subjected to extensive controls and yielded <10 colony forming units of *L. monocytogenes* per gram were released for sale. This approach was supported by further investigations that showed no growth of *L. monocytogenes* during the shelf-life of the baker’s yeast (data not shown).

Products made with baker’s yeast normally undergo a heating step that would control the contamination. However, as evident from raw dough–associated illness outbreaks caused by *Escherichia coli* and *Salmonella* in Canada and the United States (*2*,*3*), contaminated dough represents a health hazard if consumed raw, and cross-contamination can occur through the handling of contaminated baker’s yeast or contaminated raw dough. That aspect is reflected by the fact that the outbreak strain also was detected in food items in several restaurants and institutional catering establishments in different cantons (data not shown).

Listeriosis is a potentially lethal infection, and the young, the elderly, pregnant women, and immunocompromised persons are at particular risk (*4*). Foods, mainly RTE foodstuffs that include meat, fish, dairy products, fruit, and vegetables, represent major vehicles for sporadic cases and outbreaks of listeriosis (*5*). 

This outbreak, likely linked to baker’s yeast, highlights the lack of data on contamination of non-RTE products by foodborne pathogens, including *L. monocytogenes*, and the need for manufacturers of baker’s yeast to consider this risk in their production processes. Moreover, this outbreak should raise awareness that compliance with basic hygiene measures to prevent cross-contamination is particularly important when handling food that is not RTE.

Appendix Additional information about outbreak of listeriosis likely associated with baker’s yeast products, Switzerland, 2022–2024
