# Supplementary material for: Outbreak of Listeriosis Likely Associated with Baker’s Yeast Products, Switzerland, 2022–2024
Source: Emerg Infect Dis. 2024 Nov;30(11):2424–6. doi: 10.3201/eid3011.240764 (PMC11521168; doi:10.3201/eid3011.240764)
Supplement: Appendix — Additional information about outbreak of listeriosis likely associated with baker’s yeast products, Switzerland, 2022–2024 [file 24-0764-Techapp-s1.pdf]

Article DOI: https://doi.org/10.3201/eid3011.240764

EID cannot ensure accessibility for supplementary materials supplied by authors. Readers who have difficulty accessing supplementary content should contact the authors for assistance.

Outbreak of Listeriosis Likely Associated with Baker's Yeast Products, Switzerland, 2022–2024

Appendix

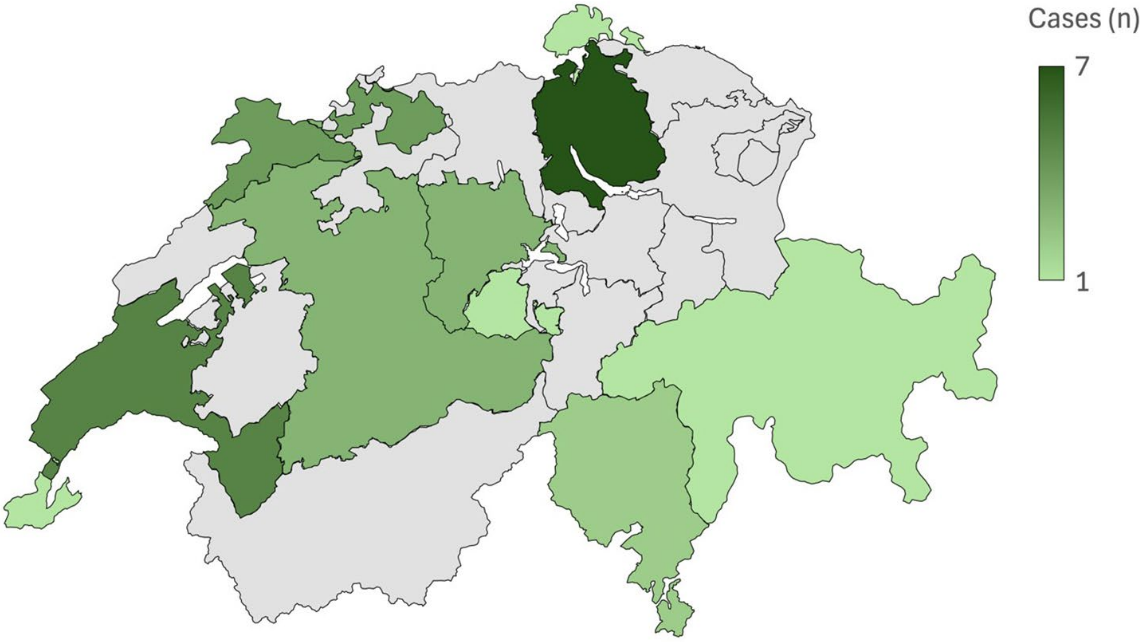

Appendix Figure. Map of Switzerland showing the cantons and the dispersion of 34 cases of human infection associated with outbreak strain *Listeria monocytogenes* serotype 1/2a-ST3141-cgMLST-CT18049 in different cantons during April 2022–June 2024. Grey, no cases recorded; shades of green, one to seven notified cases/canton.
